# Comparison of antioxidant activity in various spirulina containing products and factors affecting it

**DOI:** 10.1038/s41598-023-31732-3

**Published:** 2023-03-20

**Authors:** Agnese Stunda-Zujeva, Megija Berele, Anna Lece, Andrejs Šķesters

**Affiliations:** 1 Institute of General Chemical Engineering, Faculty of Materials Science and Applied Chemistry, Riga Technical University, Riga, Latvia; 2 Institute of Occupational Safety and Environmental Health, Scientific Laboratory of Biochemistry, Riga Stradins University, Riga, Latvia

**Keywords:** Enzymes, Chemistry

## Abstract

Spirulina is a popular food supplement known for its high antioxidant activity. Several studies have shown that antioxidant activity fluctuates depending on the combination of ingredients in the food. Fresh spirulina is a growing market trend; however, pure spirulina short shelf life is a strong limitation. This study aims to investigate antioxidant activity of various novel commercial fresh spirulina-containing products and the factors affecting it. Antioxidant activity and total phenolic content of each ingredient and binary combinations of spirulina and apple juices, Japanese quince syrup, or cranberry syrup were measured. Synergic, antagonistic, and additive interactions between samples were determined and expressed using the synergy coefficient. FRAP assay showed apparent synergism of spirulina and all the studied ingredients whereas ABTS and Folin–Ciocalteu methods revealed an antagonistic interaction between spirulina and apple juice. Despite the antagonistic interactions, all the products demonstrated at least the same antioxidant activity as pure fresh spirulina and had longer shelf life than, pointing to their commercial potential.

## Introduction

Free radicals are part of the normal metabolic process and are highly reactive. External sources like ozone, tobacco smoking, air pollutants, ionizing radiation, and industrial chemicals increase the production of free radicals. Their overproduction might lead to an imbalance between and free radicals, which, in turn, can cause damage to the biological system^1^. Many diseases (e.g., cancer, arthritis, asthma, etc.) originate from uncontrolled free radical reactions. Antioxidants neutralize free radicals, preventing cell damage and reducing the risk of potential diseases. Hence, the intake of foods rich in antioxidants is essential for optimal health. Plant foods (including algae) are the primary source of antioxidants^2^. Fast growth, high biomass productivity, and the ability to synthesize complex metabolites with minimal resources are algae’s advantages over higher plants. One of the most studied microalgae is *Arthrospira platensis* (previously known as *Spirulina platensis*) due to its high nutritional value and promising health benefits such as antioxidant, immunomodulatory, and anti-inflammatory activities.

According to the classical definition, an antioxidant is “any substance that, when present at low concentrations compared with that of an oxidizable substrate, significantly delays or inhibits oxidation of that substrate”^3^. Natural antioxidants are divided into enzymatic and non-enzymatic antioxidants^4^. Spirulina contains both groups of antioxidants. It has a high amount of SOD, GPx, CAT^5^ as well as C, E, K, and group B vitamins. It also contains β-carotene, provitamin A^6^, chlorophyll, and phycocyanin. In addition, it provides 18 amino acids, including all essential amino acids. It contains minerals such as K, Ca, Mg, Fe, Cu, Se, and Zn^7^ that can also = exhibit antioxidant properties. However, in clinical studies, the blue pigment biliprotein C-Phycocyanin is considered the main antioxidant in spirulina^8^.

Due to the above-mentioned health benefits, spirulina is gaining increasing popularity. However, a significant disadvantage reported by spirulina consumers is the specific smell and taste of spirulina powder. Therefore, a big part of spirulina is sold as pills or capsules. Currently, a new trend is quickly gaining momentum fresh frozen spirulina and dried spirulina mixed into various products to increase their nutritional value^9^. Pure fresh spirulina has a mild taste and higher nutritional value^10^; however, it has short shelf life if not frozen or dried. Therefore, new methods should be developed to increase the shelf life and improve the organoleptic properties of spirulina. Dried spirulina has positive effect on nutritional value and shelf life of yoghurt^11^, cheese^12,13^ or juice^14^.

On contrast, fruits and vegetables are common source of antioxidants and popular due to their familiar tastes. Fruits and vegetables contain a high concentration of flavonoids, one of the polyphenol groups. Berries contain diverse polyphenols that may exhibit additive, synergistic, or antagonistic interactions. These interactions may occur when different polyphenol-rich foods are ingested simultaneously^15^.

Japanese quince (*Chaenomeles japonica*) is an ornamental plant. Japanese quince fruits are rich in fibres and organic acids, mainly malic and citric acids^16^. However, due to their characteristics, such as firmness and sourness, they are not suitable for fresh consumption, so they are mainly used to produce juices, syrups, alcoholic and non-alcoholic beverages, etc.^17^. They contain antioxidants like B and C vitamins, epicatechin, iron, copper, zinc, kaempferol, quercetin, etc.^18^.

Bog cranberries (*Vaccinium oxycoccos*), also known as small cranberries, are harvested in the wild. Cranberries are rich in polyphenols such as phenolic acids, anthocyanins, and flavonoids. They are one of the few fruits with high content of proanthocyanidins, which are linked to many health benefits^19^. Bog cranberries contain antioxidants such as vitamins C, E, and K, copper, iron, malic acid, citric acid, quercetin, and catechin^19,20^.

Apples are a good source of antioxidants. They have the highest proportion of free phenolics compared to other fruits^21^. Some of the most studied antioxidants present in apples are catechin, quercetin, procyanidin, and coumaric acid^22^. Apples also contain vitamins C, A, and B and minerals like iron, zinc, calcium, magnesium, and copper^21^.

In food mixtures, the antioxidant potential is not always an additive value of all the antioxidants present in them^15^. Cömert et al.^23^ studied binary combinations of 20 foods selected from different food categories—berries, vegetables, dairy products, etc.—reporting minor to significant synergy and antagonism between those. Shi et al.^24^ mentioned that the synergistic effect of antioxidants depends on the type of antioxidants. The antioxidant activity of a binary mixture depends on the applied method, mixture composition (chemical structure and concentration), used solvent, treatment of sample, and reaction time^25,26^. Therefore, awareness of the synergy and antagonism of antioxidant capacities is crucial for developing new functional food recipes. Authors have not found studies on other ingredients effect on the shelf life an nutrition value of fresh spirulina. SIA SpirulinaNord products contains fresh spirulina and fruit syrups—both spirulina and fruits are valuable sources of antioxidants. So the aim of this study was to investigate the antioxidant activity in various commercially available fresh spirulina and berry-containing products and explore factors affecting it.

## Results

### The effect of solvent

Three solvents were compared as extractants for fresh frozen spirulina: phosphate buffered saline (PBS), 1.5% calcium chloride (CaCl_2_) aqueous solution, and ethanol. To comprehensively characterize the antioxidant activity of natural substances in the prepared extracts, ferric reducing antioxidant power (FRAP) assay, (2,2'-azino-bis(3-ethylbenzothiazoline-6-sulfonic acid)) (ABTS) assay, and total phenolic content (TPC) were applied.

**Figure 1:**
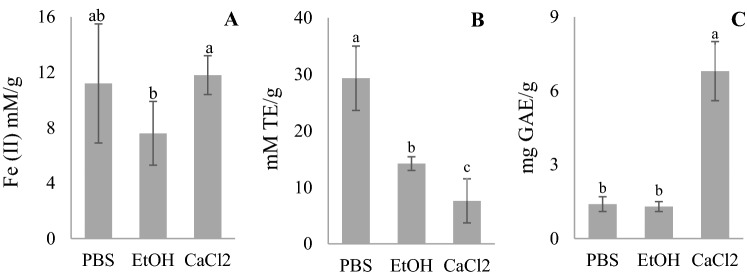
Solvent impact on fresh frozen spirulina antioxidant system activity detected by (**A**) FRAP, (**B**) ABTS, (**C**) TPC. Results expressed as (**A**) mean value ± SD Fe(II) mM/g; (**B**) mean value ± SD mM TE/g; (**C**) mean value ± SD mg GAE/g. The different letters represent significant differences at *p* < 0.05.

No significant differences (*p* > 0.05) were observed using different solvents for FRAP, while significantly different (*p* < 0.05) were the results for ABTS and TPC methods (Fig. 1). The highest results were obtained when (PBS) was used for ABTS method: 60% and 87% higher results were obtained comparing to ethanol extract and CaCl_2_ solution respectively (Fig. 1B). On the contrary, CaCl_2_ extract gave five times higher results in phenolic compounds’ content than the extracts in PBS and ethanol. (Fig. 1C).

### Spirulina in apple juice

**Figure 2:**
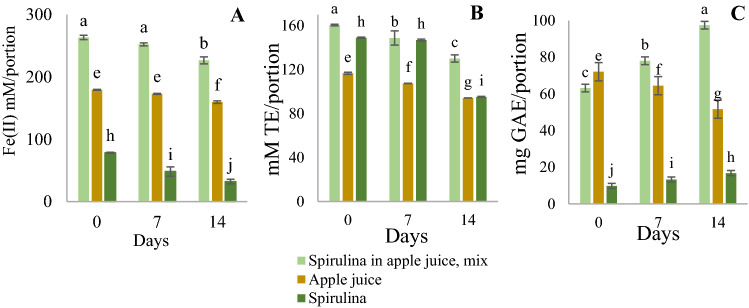
Reducing power (**A**), antioxidant activity (**B**) and polyphenol content (**C**) change with time of spirulina, apple juice, and a mixture of both at + 2… + 8 °C. The different letters for each product measurements represent significant differences at *p* < 0.05.

FRAP, ABTS, and TPC analyses were used to investigate the storage stability of apple juice, spirulina, and a mixture of both; in all cases, PBS solvent was used. After 7 days, there was a unsignificant decrease of reducing power for spirulina in apple juice and pure apple juice, while after14 days, FRAP values decreased significantly: by 14% for the mixture and 11% for apple juice (Fig. 2A). Antiradical activity decreased significantly already after seven days: by 7% (for spirulina in apple juice) and 8% (for apple juice) and 14 days, it decreased by 19% for both (Fig. 2B). Also TPC changed significantly it increased for spirulina and apple juice mixture (by 19% at seven days by 35% at 14 days), while in pure apple juice, TPC decreased by 10% and 28%, respectively (Fig. 2C). In addition, pure spirulina FRAP values decreased significantly for 38%, while ABTS value decreased only by 1% at seven days, but at 14 days to 59% and 36%, respectively (Fig. 2C). In contrast, TPC significantly increased by 26% after seven days and by 42% after 14 days. The mixture showed a negative correlation: between TPC and FRAP values, the correlation was R =  − 0.98 but between TPC and ABTS values, R =  − 0.99.

**Figure 3:**
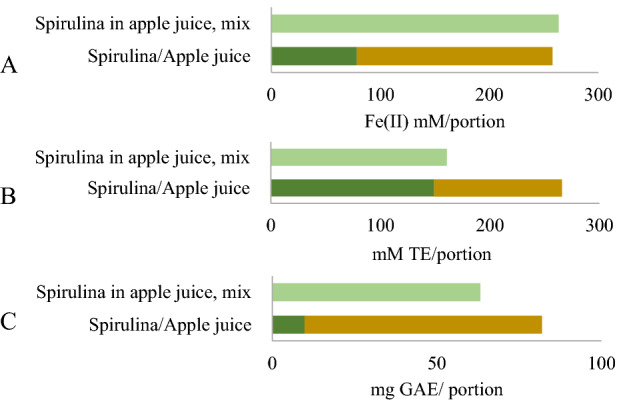
Reducing power (**A**), antioxidant activity (**B**) and polyphenol content (**C**) by portions (1st day). 7 g fresh spirulina, 14 g apple juice, and a mixture of both.

The apple juice was twice as high as that of spirulina (Fig. 3A), while higher antiradical activity was observed for spirulina (Fig. 3B). TPC was seven times higher in juice than in spirulina (Fig. 3C). The synergy effect of the antioxidant capacity of the mixtures also was differed: reducing power had additive effect (SC_FRAP_ was 1.02), while antiradical activity and TPC had antagonistic effect (SC_ABTS_ was 0.60, SC_TPC_ was 0.77).

### Spirulina in syrups

The reducing power of Japanese quince syrup was 1.9 times higher than that of the cranberry syrup, while antioxidant activity was 2.9 times higher and TPC—2.5 times higher.

**Figure 4:**
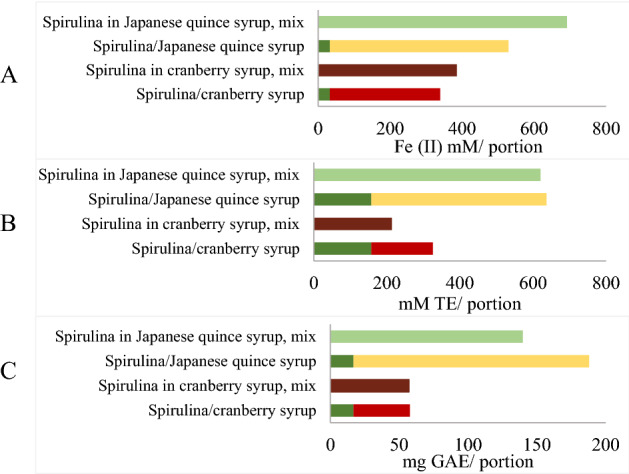
Reducing power (**A**), antioxidant activity (**B**) and polyphenol content (**C**) per portions of product (7 g fresh spirulina, 14 g Japanese quince, and cranberry syrups, and a mixture of spirulina and syrups).

There was a synergy of reducing power for spirulina in both Japanese quince syrup (SC = 1.31) and cranberry syrup (SC = 1.14) (Fig. 4A). While additive interaction for the antiradical activity was observed in a mixture of spirulina and Japanese quince syrup (Fig. 4B) and antagonism was observed in a mixture of spirulina and cranberry syrup (SC = 0.66). Opposite effects were observed by TPC, mixture with quince syrup showed antagonism (SC = 0.74), but spirulina in cranberry syrup showed additivity (SC = 1) (Fig. 4C).

## Discussion

The extraction of bioactive compounds is influenced by the solvent^27^. Since antioxidants are different kinds of chemical compounds, their solubility in different solvents varies. Three solvents were compared in this study. As phycocyanin is the main antioxidant of spirulina, giving various health benefits, common solvents for phycocyanin extraction were used: (1) an aqueous solution of phosphate-buffered saline (PBS); (2) calcium chloride (CaCl_2_) and ethanol^28–31^. Ethanol extracts of spirulina have higher total phenolic and flavonoid content and higher carotenoid and chlorophyll concentrations compared to aqueous extracts^32,33^. Aqueous extracts have higher concentrations of phycocyanin, free amino acids, carbohydrates, and total protein than spirulina ethanol extracts. However, they have lower antioxidant activity in the ABTS method^34^.

No significant difference between PBS, CaCl_2_ aqueous solution, and ethanol extracts was found in FRAP assay (Fig. 1A). Although FRAP assay is widely used for spirulina characterization, it cannot detect protein antioxidant activities, according to the literature^35^. Therefore, phycocyanin, a phycobiliprotein antioxidant activity, cannot be demonstrated by FRAP but only the activity of the antioxidants that can reduce the Fe^3+^ ion in acidic conditions, such as vitamins C and E and polyphenols^36^.

The solvent significantly influenced the antiradical activity (ABTS method) of fresh frozen spirulina biomass (*p* = 0.01). Higher antiradical activities were obtained if more polar solvents (water-based extracts) were used (Fig. 1B). The other disadvantage of alcohol extracts is longer reaction times^37^—more than 30 min were required. The reactions of the ABTS method can take place both through the hydrogen atom transfer mechanism (HAT) and the single electron transfer mechanism (SET); they occur in neutral pH media or close to it. All the antioxidants that can scavenge the ABTS radical cation during the reaction are involved^38^.

CaCl_2_ water solution showed several times higher total phenolic content than ethanol, which can be explained by the higher polarity of the extractant favourable for polyphenolic compound extraction. On the other hand, PBS showed lower TPC than CaCl_2_ solution because of disturbing ions present in the PBS solution. Phycocyanin is insoluble in ethanol but soluble in water^39^. Therefore, extracts in ethanol could be assumed as phycocyanin-free extracts and should show lower antioxidant activity (Fig. 1). PBS water solution showed similar results to ethanol extract for TPC content, as phycocyanin is not a phenolic compound and, thus, is not detected by the TPC method. Solvent significantly influenced TPC (*p* < 10^−6^). The difference between PBS and CaCl_2_ water solution in ABTS could be due to the fact that a lower yield of phycocyanin is obtained with aqueous CaCl_2_ than with PBS. Since CaCl_2_ dissolves only sodium-calcium channels in spirulina cells. At the same time, PBS damages the whole cell^30,40^. Therefore, it was decided to use PBS water solutions further in this study.

Similarly, significant differences between both solvents and methods were observed by other authors. Shukla et al.^41^ determined spirulina antioxidant activity with two assays—FRAP and ABTS. While FRAP values for water and ethanolic extracts were 0.09 and 0.25 mM TE/L, ABTS values were 1.33 and 1.73 mM TE/L. Safari et al.^42^ studied phycocyanin antioxidant activity using PBS as a solvent. They determined that ferric reducing antioxidant power was only 0.05 mg TE/g, but DPPH showed more than 45% inhibition indicating phycocyanin as a potent radical scavenger. Factors such as stereoselectivity of the radicals, mechanism of action, and the solubility of the extract in different testing systems may also affect the capacity to scavenge different radicals^34^.

Fresh spirulina has a high moisture content, and it is considered a perishable product. The decrease of spirulina antioxidant activity (Fig. 2A) in seven days is not significant (*p* > 0.05), hence it could be used within seven days after it had been thawed. However, the organoleptic properties of the product were worsening—the specific aroma appeared. In contrast the shelf life of spirulina in apple juice is 3 months. The reducing power of spirulina and apple juice mixture within 14 days decreased by less than 15% (Fig. 2B). The synergic effect was not detected on the 0 day, while it appeared on the 7th and 14th days. Within 14 days, ABTS values dropped by less than 20%, and organoleptic properties (colour, taste, and aroma) did not practically change.

While reducing power and antioxidant activity decreased, the content of polyphenolic compounds in spirulina samples and spirulina with apple juice increased with increasing storage time. In contrast, polyphenols content of pure apple juice decreased during storage time (Fig. 2C). Figure 2C demonstrates that TPC and FRAP of apple juice had approximately 8 times higher than TPC of spirulina. While the FRAP value of apple juice was two times higher than spirulina, the ABTS value was 1.3 times lower than spirulina. This indicates that the antioxidant activity of apple juice is mainly due to the polyphenolic compounds. However, spirulina has a complicated composition that is relatively high in proteins; thus, it could be suggested that interaction between different classes of chemical compounds led to the formation of various degradation products and derivatives which reacted with Folin–Ciocalteu reagent, besides phenols. As a result, it showed higher TPC value^43^. The increase in TPC and decrease in antioxidant activity led to a negative correlation.

Furthermore, the spirulina and apple juice mixture showed a negative correlation: between TPC and FRAP values, the correlation was R =  − 0.98 while between TPC and ABTS values, it was R =  − 0.99. A negative correlation indicates that the antioxidant activity of spirulina and apple juice mixture during storage in a refrigerator does not depend only on phenolic content.

The synergy analysis of spirulina in apple juice showed an antagonistic effect for ABTS and TPC and only a slightly positive one for FRAP (Fig. 3). However, a positive commercial aspect is that spirulina in apple juice has at least the same antioxidant and antiradical properties as frozen spirulina while it can be stored in a refrigerated, not frozen state.

The synergy analysis of spirulina in cranberry or Japanese quince syrup showed both synergistic and antagonistic effects. As summarized in (Table 1), FRAP shows synergy (SC > 1) for all the products, while ABTS and TCP results showed antagonistic effects (SC < 1). Similar effects were found in other studies. The analysis of SC calculated data in^23^ showed that when using water as a solvent, the ABTS results of mixtures can give up to 3 times lower or higher antioxidant activity than the sum of both ingredients. The highest synergy was observed for wine with black grapes, followed by milk with black tea. Black grapes, black tea, and espresso with fermented milk products currently dominate the synergy TOP list. As fruits and seeds contain phenols, milk protein reactions with phenols may be the reason of antagonism^44^. Protein sources such as milk and beans create an antagonistic effect for berries. The only protein sources showing synergy with various berries were yoghurt and Adzuki beans.

**Table 1:** Synergy coefficients of spirulina containing products.

Product	Coefficient		
	SC_FRAP_	SC_ABTS_	SC_TPC_
Spirulina + Apple juice	1.02	0.60	0.77
Spirulina + Japonica Quince syrup	1.31	0.97	0.74
Spirulina + Cranberry syrup	1.14	0.66	0.99

Spirulina contains 60–70% protein of dry weight^7^; therefore, in a mixture with fruits containing polyphenols, the antagonistic effect could be explained. The summary of synergy effects is shown in Table 1. Absolute units given in the study are converted to dimensionless synergy coefficient (SC).

FRAP assay showed the most positive antioxidant interactions, while ABTS demonstrated neutral or antagonistic effects. Comparing the obtained values (Table 1), spirulina and apple juice showed significant antagonism in antiradical activity for the ABTS method—SC was 0.60, while the most profound antagonism effect described in^23^ was for milk with breakfast cereal (SC_ABTS_ 0.35), chia seed (0.44) and flaxseed (0.55) (see Fig. 4). One of the pieces of evidence of antagonism may be an increase in polyphenolic compounds when samples are stored in a refrigerator (refer to Fig. 2), indicating that chemical and structural changes had occurred. These results indicate that each method observes different aspects of the antioxidant system. Although, the mixture of products forms a new system of antioxidants, some of the substances had interacted, changing the overall antioxidant/antiradical activity.

However, from a consumption perspective, all the supplements preserved or improved the antioxidant activity of the product compared to spirulina alone. Even in cases of antagonistic interaction, spirulina products had the same or higher antioxidant activity compared to that of fresh spirulina alone. The additive effect of reducing capacity showed that consuming spirulina alone will give 3 times less Fe^3+^ ion-reducing antioxidants than if it is consumed in a mixture with apple juice.

The reducing power in a mixture with Japanese quince was 21 times higher than that of spirulina alone, but in cranberry syrup it was 12 times higher. For ABTS assay, spirulina in Japanese quince syrup showed an additive effect while in cranberry syrup—antagonistic effect. Both mixtures showed higher activity than spirulina alone (4 times in Japanese quince and 1.4 times in cranberry syrup). FRAP had a bigger difference between a binary mixture and pure spirulina than the ABTS assay. As previously mentioned, this happens because FRAP does not detect protein (phycocyanin) antioxidant activity.

Total phenolic content in a binary mixture with spirulina and Japanese quince syrup showed antagonistic interaction, but cranberry syrup showed an additive effect. Sugar may cause a reduction in the antioxidant activity of the polyphenols. Condensation reactions can occur between sugar molecules and polyphenols. As a result of these reactions, glycosides like pentagalloylglucose and tetragalloyglucose are likely to be formed. However, sucrose molecules can interact with oxidized phenolic compounds. It would result in the formation of the reduced form of phenolic compounds and increase antioxidant activity^45^.

Similar to spirulina and apple juice mixture, spirulina in syrups showed a negative correlation. Spirulina in cranberry syrup correlation was R =  − 0.95 (between TPC and FRAP) and R =  − 0.92 (between TPC and ABTS). Spirulina in Japanese quince syrup correlation was R =  − 0.90 and R =  − 0.98. The correlation in the researched mixtures showed that antioxidant activity does not depend only on polyphenols. Antioxidant activity decreases while polyphenolics increase.

Considering food as a source of antioxidants for human health, our study shows that fresh spirulina antioxidant activity is considerably lower than that of Japanese quince or cranberry syrups. The antioxidant capacity of Japanese quince and cranberries is largely determined by polyphenols and similar compounds that are not fully absorbed by the human body, while spirulina contains various types of compounds that are beneficial to the body, including those with antioxidant activity. Clinical studies^46,47^ highlight C-phycocyanin as the most valuable antioxidant. At the same time, spirulina contains fewer polyphenols than fruits or berries.

Future antioxidant studies of spirulina-containing products should be continued by also applying other methods, as well as by setting the objective to investigate the actual effect of antioxidants.

## Materials and methods

### Samples and chemicals

The research was performed on commercial products containing microalgae *Arthrospira platensis* (hereinafter Spirulina). Commercial samples were provided by SIA “SpirulinaNord”: (i) spirulina—50% fresh spirulina and 50% water; (ii) spirulina in apple juice—32% fresh spirulina and 68% apple juice; (iii) spirulina in cranberry syrup—32% fresh spirulina, 34% cranberry juice, and 34% sugar; (iv) spirulina in Japanese quince syrup—32% fresh spirulina, 43% Japanese quince juice, and 25% sugar. The samples were frozen and kept before analysis at (− 20 °C).

Shot storage stability studies were conducted with thawed samples of spirulina and spirulina with apple juice stored in a refrigerator (2–8 °C) for 14 days; the analyses were performed on the defrosting day (0 day), on the 7th day and on the 14th day after melting.

Results for mixtures were calculated for one portion (21 g), which consists of 7 g of spirulina (without water) and 14 g of juice or syrup.

Three extractants (ethanol, phosphate-buffered saline (PBS) standard solution, 1.5% CaCl_2_ aqueous solution) were used for extract preparation, diluting thawed samples 25 times.

2,2'-azino-bis(3-ethylbenzothiazoline-6-sulfonic acid) (ABTS), Trolox, gallic acid, Folin-Ciocalteu reagent, 2,4,6-tripyridyl-s-triazine (TPTZ), calcium chloride, iron salts, sodium salts, potassium persulfate were purchased from Sigma-Aldrich. PBS tablets were purchased from Thermo Fisher. All chemicals and reagents were of analytical grade and were used as received without further purification. Absorbance measurements were performed using a UV–Vis Varian Cary 50 spectrophotometer.

Various analytical methods were used to assess the antioxidant activity of samples.

### Ferric reducing antioxidant power (FRAP)

Ferric reducing antioxidant power assay was performed according to the method described by Benzie and Strain^48^. The FRAP reagent was always freshly prepared by mixing 300 mM acetate buffer (pH 3.6), 10 mM TPTZ dissolved in 40 mM HCl, and 20 mM FeCl_3_·6H_2_O solution in 10:1:1 ratio. Three millilitres of freshly prepared working FRAP reagent were mixed with 0.2 mL of the sample extract. The mixture was incubated at 37 °C for 5 min. The absorbance was measured at 593 nm against a reagent blank. The FRAP value was calculated and expressed as mM Fe(II) × g^−1^ of product based on a calibration curve plotted using an aqueous solution of 1 mM ferrous sulphate (FeSO_4_·7H_2_O).

### ABTS radical scavenging assay

Extensively reported ABTS radical cation decolorization assay was performed according to the method by Re et al.^49^. ABTS^●+^ was produced by mixing a 7 mM stock solution with 2.45 mM potassium persulfate. The mixture was kept in the dark at room temperature for 16 h before use. The ABTS^●+^ solution was diluted with methanol to achieve an absorbance of 0.700 ± 0.025 nm. Once the mentioned absorbance reached 2.97 mL of ABTS^●+^, the solution was mixed with 0.03 mL of sample extract and incubated at 37 °C. Absorbance was measured after 6 min of incubation. Trolox was used as the reference standard, and the results were expressed as mM TE × g^−1^ of the product.

### Total phenolic compounds (TPC)

The widely used Folin-Ciocalteu method was applied for the determination of total phenolic compounds (TPC) using the procedure described by Blainski et al.^50^. To carry out the analysis, 1.5 mL 10% Folin–Ciocalteu reagent was added to 0.25 mL sample extract, mixed and left in the dark at room temperature for 5 min; then 1 mL 7.5% sodium carbonate solution was added to the mixture and kept at room temperature for 30 min in the dark. The absorbance measurement was performed at 765 nm. Gallic acid was used as a reference standard, and the results were expressed as mg GAE × g^−1^ product.

### Synergy coefficient (SC)

For synergy effect characterization, we introduced the *synergy coefficient (SC)* that was calculated as a ratio between experimentally detected antioxidant activity and calculated antioxidant activity (the sum of antioxidant activity of each ingredient). The coefficient was SC > 1 in case of synergy, and SC < 1 in case of antagonism, SC = 1 in case of additivity meaning antioxidant activity for mixture as the sum of antioxidant activity of pure ingredients.

### Calculations

The experiment was done in duplicate for each extract. Readings for 1 g of sample were calculated according to the following equation:

$$ {\text{C}}_{2} = ({\text{C}}_{1} \times {\text{V}})/{\text{m}}$$

where C_2_ is concentration in the sample, C_1_ is concentration in the sample after calibration curve, V is the volume of the extract, and m is the mass of the sample in the extract. All data were expressed as mean value ± standard deviation.

### Statistical analysis

Data were analysed by one-way analysis of variance (ANOVA). Differences were considered significant at *p* < 0.05. The relationship between the concentration of Total Phenolic compounds and antioxidant activity were described using correlation bivariate statistic. A direct correlation between TPC and antioxidant activity was determined by linear regression analysis. Trend line equations were estimated according to coefficient of determination (R^2^).

## Conclusions

With the development of the functional food market, interactions between various food ingredients have become a topic of major interest. In this study, the antioxidant capacity of new commercial products was studied and compared to the antioxidant properties of single ingredients. Antioxidant properties of ethanol and water-based (PBS and CaCl_2_) extracts of spirulina differed significantly for ABTS and TPC (*p* < 0.05) while no significant difference was observed for FRAP assay. PBS extracts of fresh frozen spirulina and products containing fresh spirulina were compared using FRAP, ABTS, and TPC methods. The synergy coefficient was introduced to characterize the result of mixtures interaction, dimensionless. Antioxidant synergy was observed in all samples for FRAP method, while additive effect or antagonism were observed by ABTS and TCP, which could be due to the complex nature of antioxidant systems interaction and each method’s inability to describe it fully. However, from a consumption perspective, all the studied additives preserved or improved the antioxidant activity of the product, compared to spirulina alone, and extended the shelf life of fresh spirulina.

## Data Availability

The datasets used and/or analyzed during the current study available from the corresponding author on reasonable request.

## References

[1] Lobo V, Patil A, Phatac A, Chandra N (2010). Free radicals’ antioxidants and functional foods impact human health. Pharmacogn. Rev..

[2] Carlsen MH (2010). The Total antioxidant content of more than 3100 foods, beverages, spices, herbs and supplements used worldwide. Nutr. J..

[3] Halliwell B, Gutteridge JMC (1995). The definition and measurements of antioxidants in biological systems. Free Radical Biol. Med..

[4] Irato P, Santovito G (2021). Enzymatic and non-enzymatic molecules with antioxidant function. Antioxidants.

[5] Asghari A, Fazilati M, Latifi AM, Salavati H, Choopani A (2016). A review on antioxidant properties of spirulina. J. Appl. Biotechnol. Rep..

[6] Romay C, Gonzalez R, Ledon N, Remirez D, Rimbau V (2003). C-phycocyanin: A biliprotein with antioxidant, anti-inflammatory and neuroprotective effects. Curr. Protein Pept. Sci..

[7] Jung F, Kruger-Genge A, Waldeck P, Kupper JH (2019). Spirulina platensis, a super food?. J. Cell. Biotechnol..

[8] Finamore A, Palmery M, Bensehaila S, Peluso I (2017). Antioxidant, immunomodulating, and microbial-modulating activities of the sustainable and ecofriendly *Spirulina*. Oxid. Med. Cell. Longev..

[9] Distribution Channel. Spirulina market. *Mericulous Research*. Spirulina Market by Size, Share, Forecasts, & Trends Analysis (meticulousresearch.com) (2021).

[10] Ma Z (2019). Fresh living Arthrospira as dietary supplements: Current status and challenges. Trends Food Sci. Technol..

[11] Patel P (2019). Development of a carotenoid enriched probiotic yogurt from fresh biomass of *Spirulina* and its characterization. J. Food Sci. Technol..

[12] Agustini TW, Maruf WF, Widajat W, Suzery M, Hadijanto H, Benjakul S (2016). Application of *Spirulina platensis* on ice cream and soft cheese with respect to their nutritional and sensory perspectives. J. Teknologi.

[13] Golmakani MT, Zad SS, Alavi N, Nazari E, Eskandari MH (2019). Effect of Spirulina (*Arthrospira platensis*) powder on probiotic bacteriologically acidified feta-type cheese. J. Appl. Phycol..

[14] Wajda L (2020). Dried biomass of *Arthrospira platensis* inhibits growth of *Aureobasidium pullulans* LW14 and some bacteria when added to unpasteurised apple juice. Indian J. Microbiol..

[15] Wang S, Meckling KA, Marcone MF, Kakuda Y, Tsao R (2011). Synergistic, additive, and antagonistic effects of food mixtures on total antioxidant capacities. J. Agric. Food Chem..

[16] Czubinski J, Ruško J, Gornas P (2021). Japanese quince seeds as a promising rich source of proteins and essential amino acids. Plant Food Hum. Nutr..

[17] Urbanavičiūtė, I., Viškelis, P. Biochemical composition of Japanese quince (*Chaenomeles japonica*) and its promising value for food, cosmetic, and pharmaceutical industries in *Fruit Industry [Working Title]* (ed. Kahramanoglu, I.) 1–24 (IntechOpen, 2022).

[18] Bosiacka IB (2017). Macro- and microelement content and other properties of *Chaenomeles japonica* L. fruit and protective effects of its aqueous extract on hepatocyte metabolism. Biol. Trace Elem. Res..

[19] Nemzer BV, Al-Taher F, Yashin A, Revelsky I, Yashin Y (2022). Cranberry: Chemical composition, antioxidant activity and impact on human health: Overview. Molecules.

[20] Česoniene L, Daubaras R, Areškevičiūte J, Viškelis P (2006). Evaluation of morphological peculiarities, amount of total phenolics and anthocyanins in berries of European cranberry (*Oxycoccus palustris*). Balt. For..

[21] Lee CY (2012). Common nutrients and nutraceutical quality of apples. N. Y. Fruit Q..

[22] Boyer J, Liu RH (2004). Antioxidants of apples. N. Y. Fruit Q..

[23] Comert ED, Gokmen V (2022). Effect of food combinations and their co-digestion on total antioxidant capacity under simulated gastrointestinal conditions. Curr. Res. Food Sci..

[24] Shi J, Qu Q, Kakuda Y, Xue SJ, Jiang Y, Koide S, Shim YY (2007). Investigation of the antioxidant and synergistic activity of lycopene and other natural antioxidants using LAME and AMVN model systems. J. Food Compos. Anal..

[25] Sonam KS, Guleria S (2017). Synergistic antioxidant activity of natural products. Ann. Pharmacol. Pharm..

[26] Hidalgo M, Sanchez-Moreno C, Pascual-Teresa S (2010). Flavonoid–flavonoid interaction and its effect on their antioxidant. Food Chem..

[27] Rebey IB, Bourgou S, Debez IBS, Karoui IJ, Sellami IH, Msaada K, Limam F, Marzouk B (2012). Effects of extraction solvents and provenances on phenolic contents and antioxidant activities of cumin (*Cuminum cyminum* L.) Seeds. Food Bioprocess Technol..

[28] Dianursanti H, Aini M (2021). The enhancement of phycocyanin yield and purity from Spirulina platensis using freeze-thawing method on various solvent. AIP Conf. Proc..

[29] Prabuthas P, Majumdar S, Srivastav PP, Mishra HN (2011). Standardization of rapid and economical method for neutraceuticals extraction from algae. J. Stored Prod. Postharvest Res..

[30] Ilter I, Akyil A, Demirel Z, Koc M, Conk-Dalay M, Kaymak-Ertekin F (2018). Optimization of phycocyanin extraction from Spirulina platensis using different techniques. J. Food Compos. Anal..

[31] Zavrel T, Chmelik D, Sinetova MA, Cerveny J (2018). Spectrophotometric determination of phycobiliprotein content in cyanobacterium synechocystis. J. Vis. Exp..

[32] Rutar JM, Cillero-Pastor B, Monhren R, Ulrih NP, Orgrinc N, Jamnik P (2021). Insight into antioxidant effect of fermented and non-fermented spirulina water and ethanol extracts at the proteome level using a yeast cell model. Antioxidants.

[33] Gabr GA, El-Sayed SM, Hikal MS (2020). Antioxidant activities of phycocyanin: A bioactive compound from Spirulina platensis. J. Pharm. Res. Int..

[34] Chu WL, Lim YW, Radhakrishnan AK, Lim PE (2010). Protective effect of aqueous extract from Spirulina platensis against cell death induced by free radicals. BMC Complement. Altern. Med..

[35] Ioannou, I., Chaaban, H., Slimane, M., Ghoul. Origin of the variability of the antioxidant activity determination of food material, in *Biotechnology* (ed. Ekinci, D.) 77–92 (IntechOpen, 2015).

[36] Benzie, F.F.I., Devaki, M. The ferric reducing/antioxidant power (FRAP) assay for non-enzymatic antioxidant capacity: concepts, procedures, limitations and applications, in *Measurement of Antioxidant Activity & Capacity* (ed. Apak, R., Capanoglu, E., Shahidi, F.) 77–106 (John Wiley & Sons, 2017).

[37] Santos-Sánchez, N.F., Salas-Coronado, R., Villanueva-Cañongo, C., Hernández-Carlos, B.Antioxidant compounds and their antioxidant mechanism, in *Antioxidants* (ed. Shalaby, E.) 1–28 (IntechOpen, 2019).

[38] Shahidi F, Zhong Y (2015). Measurement of antioxidant activity. J. Funct. Foods.

[39] Liu Q, Huang Y, Zhang R, Cai R, Cai Y (2016). Medical application of spirulina platensis derived C-phycocyanin. J. Evid. Based Complement. Altern. Med..

[40] Rajmohan D, Bellmer D (2019). Characterization of Spirulina-alginate beads formed using ionic gelation. Int. J. Food Sci..

[41] Shukla V, Vashistha M, Singh SN (2009). Evaluation of antioxidant profile and activity of amalaki (*Emblica officinalis*), spirulina and wheat grass. Indian J. Clin. Biochem..

[42] Safari R, Raftani AZ, Esmaeilzadeh KR (2020). Antioxidant and antibacterial activities of C-phycocyanin from common name *Spirulina platensis*. Iranian J. Fish. Sci..

[43] Everette JD, Bryant QM, Green AM, Abbey YA, Wangila GW, Walker RB (2010). Thorough study of reactivity of various compound classes toward the Folin–Ciocalteu reagent. J Agric. Food Chem..

[44] Bandyopadhyay P, Ghosh AK, Ghosh C (2012). Recent developments on polyphenol–protein interactions: Effects on tea and coffee taste, antioxidant properties and the digestive system. Food Funct..

[45] Shalaby EA, Mahmoud GI, Shanab SMM (2016). Suggested mechanism for the effect of sweeteners on radical scavenging activity of phenolic compounds in black and green tea. Front. Life Sci..

[46] Deng R, Chow TJ (2010). Hypolipidemic, antioxidant and anti-inflammatory activities of microalgae *Spirulina*. Cardiovasc. Ther..

[47] Karkos PD, Leong SC, Karkos CD, Sivaji N, Assimakopoilos DA (2010). Spirulina in clinical practice: Evidence-based human applications. Evid. Based Complement. Altenat. Med..

[48] Benzie FF, Strain JJ (1999). Ferric reducing/antioxidant power assay: Direct measure of total antioxidant activity of biological fluids and modified version for simultaneous measurements of total antioxidant power and ascorbic acid concentration. Methods Enzymol..

[49] Re R, Pellegrini N, Proteggente A, Pannala A, Yang M, Rice-Evans C (1999). Antioxidant activity applying an improved ABTS radical cation decolorization assay. Free Radical Biol. Med..

[50] Blainski A, Lopes GC, Mello JCP (2013). Application and analysis of the Folin Ciocalteu method for the determination of the total phenolic content from *Limonium brasiliense* L. Molecules.

